# Liposome mediated double-stranded RNA delivery to silence ribosomal protein P0 in the tick *Rhipicephalus haemaphysaloides*

**DOI:** 10.1016/j.ttbdis.2018.01.015

**Published:** 2018-03

**Authors:** Yuting Zhang, Jie Cui, Yongzhi Zhou, Jie Cao, Haiyan Gong, Houshuang Zhang, Jinlin Zhou

**Affiliations:** Key Laboratory of Animal Parasitology of Ministry of Agriculture, Shanghai Veterinary Research Institute, Chinese Academy of Agricultural Sciences, Shanghai 200241, China

**Keywords:** Tick, Liposome, dsRNA delivery, P0 gene, Anti-tick agents

## Abstract

Control of ticks has been achieved primarily by the application of acaricides, which has drawbacks such as environmental contamination leading to the selection of pesticide-resistant ticks. The potential of dsRNA to suppress genes critical for tick survival due to its sequence specificity suggests that dsRNAs could be developed as tailor-made pesticides. In this study, the dsRNA of P0 gene from the tick, *Rhipicephalus haemaphysaloides*, was evaluated as a potential anti-tick agent. Effects of using different dsRNA delivery methods were tested by quantitative RT-PCR and tick bioassays to determine survival, feeding and reproduction. The results showed that P0 dsRNAs could be effectively delivered into ticks and silenced by incubating with liposomes. Incubation time was found to be the most important factor in dsRNA delivery and gene silencing compared with liposome types and dsRNA concentration. The effects of P0 dsRNA treatment on ticks were found to be significant on blood feeding, molting or reproduction. These data show that anti-tick agents based on dsRNAs could have potential use in tick control.

## 1 Introduction

Ticks are obligate hematophagous ectoparasites of wild and domestic animals as well as humans. They are considered to be second only to mosquitoes as global vectors of human diseases (de la Fuente et al., 2008; Jongejan and Uilenberg, 2004). Control of ticks has been achieved primarily by the application of acaricides, a method that has drawbacks such as environmental contamination and selection of pesticide-resistant ticks. These issues reinforce the need for alternative approaches to control tick infestations (de la Fuente and Kocan, 2006). RNA interference (RNAi), which is the sequence-specific degradation of mRNA mediated by homologous double-stranded (dsRNA), has become a valuable tool in gene knockdown in eukaryotes (Fire et al., 1998; Yu et al., 2013a). The potential of dsRNA to suppress genes critical for insect survival due to its sequence specificity suggests that dsRNAs could be developed as tailor-made pesticides (Huvenne and Smagghe, 2010; Zhang et al., 2013).

dsRNA-mediated gene silencing has been extensively used for the analysis of gene functions in ticks. Particularly, long dsRNAs have been routinely applied successfully in many tick species for targeted gene knockdown in various stages of tick lifecycle, with evidence of systemic RNAi spread into subsequent stages (de la Fuente et al., 2007; Karim and Adamson, 2012). The main goal of this method is to study tick physiology and to discover new targets to control. Notably, sterile ticks were created by knocking down a molecule named subolesin using RNAi. Consequently, the release of subolesin-silenced ticks was proposed as a sterile acarine technique (SAT) for autocidal control of tick populations (de la Fuente et al., 2006). The efficacy of SAT with dsRNA has been demonstrated to control tick infestations alone or in combination with subolesin vaccination, although some limitations have to be considered when large numbers of subolesin-knockdown ticks will be released into the environment (Merino et al., 2011).

The application of RNAi-triggering molecules as therapeutic or control agents has been progressing in mammals and insects, although in ticks progress in this subject has been slow. There are many limitations in using RNAi-based technologies for tick control; selection of the target gene and reliable double-strand RNA (dsRNA) delivery are the two major challenges. For example, micro-injection, electroporation, and artificial capillary feeding, which are methods to introduce dsRNA in ticks, can only be successfully used in laboratory experiments (Karim et al., 2010; Karim and Adamson, 2012; Ruiz et al., 2015). Soaking tick tissues or cells in dsRNA has been reported (de la Fuente et al., 2007). A recent report indicated the effectiveness of immersing whole ticks in dsRNA to induce gene silencing (Galay et al., 2016). Although naked dsRNA can be internalized at high concentration in sterile conditions in the laboratory, dsRNA in field settings could be subject to rapid environmental degradation (Dubelman et al., 2014; Fischer et al., 2017). To prevent RNAi trigger degradation, abiotic and biotic delivery systems including the use of liposomes, chitosan nanoparticles, *E. coli* expression systems, and *Pichia pastoris* expression systems have been explored in insects (Whyard et al., 2015; Van Ekert et al., 2014, Zhang et al., 2010; Bedoya-Perez et al., 2013). However, there are no such reports for tick dsRNA delivery systems.

A previous study suggested that gene silencing of the ribosomal protein, P0, was lethal to the tick, *Haemaphysalis longicornis* (Gong et al., 2008). Administering anti-P0 protein in *Rhipicephalus* spp. ticks resulted in high mortality and poor egg hatch rate (Rodríguez-Mallon et al., 2012). These results indicated that the P0 gene could be used as the key target for killing ticks and decreasing reproduction.

This study focused on investigating the best method to deliver P0 dsRNAs *via* the surface absorption using different liposomes under different conditions in order to develop a novel anti-tick biological agent.

## 2 Materials and methods

### 2.1 Tick collection and maintenance

A colony of *R. haemaphysaloides* ticks was initiated from one engorged female collected from a buffalo in Hubei, China (Zhou et al., 2006). After three generations of rearing under laboratory conditions, the tick colony was established in the Shanghai Veterinary Research Institute, Chinese Academy of Agricultural Sciences, Shanghai, China. Ticks were reared in a dark incubator at 25 °C with 92% relative humidity and fed on a New Zealand white rabbit. Larvae, nymphs and adult ticks were collected for experimental use.

### 2.2 Cloning and sequence analysis of *R. haemaphysaloides* P0 gene

An expressed sequence tag (EST) of the *R. haemaphysaloides* P0 gene was found in the salivary gland cDNA library previously constructed in our laboratory (Yu et al., 2015). To clone the full-length cDNA of the *R. haemaphysaloides* P0 gene, 3′ and 5′ RACE was used according to the manufacturer’s instructions (Invitrogen, Life Technologies Corporation, USA). Amplified PCR fragments were cloned into the pEGM-T vector, and the nucleotide sequences of the positive clones were sequenced. The obtained full-length cDNA sequence of ribosomal P0 was then analyzed.

### 2.3 Preparation of dsRNAs

The P0 cDNA in the pGEM-T vector was amplified using PCR primers 5′-GGATCCTAATACGACTCACTATAGGCTTCAACTTCCTTTCATACGG-3′ and 5′-GGATCCTAATACGACTCACTATAGGCACCCTCAACGAATGCTG-3′ containing the T7 promoter sequence at their 5′ ends. In addition, the cDNA fragment encoding bacterial luciferase (Yu et al., 2013b) was also amplified using the primers 5′-GGATCCTAATACGACTCACTATAGGGCTTCCATCTTCCAGGGATACG-3′ and 5′-GGATCCTAATACGACTCACTATAGGCGTCCACAAACACAACTCCTCC-3′ containing the T7 promoter sequence at their 5′-ends. Luciferace dsRNA was used as the negative control. The PCR products were gel-purified and RNA was synthesized by *in vitro* transcription using the T7 RiboMAXTM Express RNAi System (Promega, USA). Then, dsRNAs were quantified by spectrometry at 260 nm, and their integrity was checked on 1% agarose gels, and stored at −20 °C until further use.

### 2.4 Real-time PCR assay for gene silence

Total RNA was extracted from *R. haemaphysaloides* using the RNeasy Mini Kit (QIAGEN, Germany) following the manufacturer’s instructions. cDNA was synthesized from the total RNA using the Takara RNA PCR Kit (AMV) Ver. 3.0 (Takara Bio Inc. Japan). SYBR green real-time PCR amplifications were performed using 0.1 μg of cDNA and oligonucleotide primers specific for P0 in a final volume of 20 μl. Transcript abundance was measured using a Light Cycler 1.5 (Roche Instrument Center AG, Roikreuz, Switzerland) according to the manufacturer’s instructions. The tick elongation factor 1α gene was used as an internal control (Nijhof et al., 2007). The 2^−△△CT^ method was used to calculate the relative expression levels of P0. Each analysis was repeated three times. The primers used for real-time PCR are as follows: 5′-TTCTCGTATGGTCTGAAGATTTT-3′ and 5′-AACAGTTGGGTATCCGATGG -3′ for the P0 gene, and 5′-CGTCTACAAGATTGGTGGCATT-3′ and 5′-CTCAGTGGTCAGGTTGGCAG-3′ for the elongation factor-1α gene.

### 2.5 Testing P0 gene silencing efficiency

Three kinds of lipid agents, Lipofectamine 2000, DMRIE-C and Cellfectin (Invitrogen, Life Technologies Corporation, USA) were tested in this study. Based on the concentrations suggested in the manufacturer’s instructions for DNA and RNA transfections, we used 1 μg P0 dsRNA/μl liposome solution (the proportion of water and liposome was 1:1) as the test dsRNA concentration and soaked for 24 h. For the control group, 1 μg P0 dsRNA/μl RNase free water was used. To calculate the rate of relative expression, ticks soaked in water without P0 dsRNA were used as the control. Larvae, nymphs and adult ticks were all tested. In soaking tests, ∼100 larvae, 50 nymphs, or 20 adults were placed into 2 ml tubes, to which 1 ml dsRNA with liposome or control dsRNA was added. Ticks were soaked by setting the tube on a vertical rotating device at room temperature for 24 h. Then, ticks were rinsed in distilled water and dried on paper towels. Afterward they rested for 5 d before RNA was extracted for real-time PCR analysis. Relative expression of the P0 gene against the tick elongation factor-1 was calculated using the 2^−△△CT^ method, and then the rate of relative mRNA expression against the control (ticks soaked in water without P0 dsRNA as 100% relative expression) was calculated for final results. Each test was repeated three times.

### 2.6 Observation of Cy3-labeled P0 dsRNA in *R. haemaphysaloides* after soaking

To observe the delivery of dsRNA into the tick body, the P0 dsRNA was labeled using the CyTM3 Silencer siRNA labeling kit (Ambion Thermo Fisher Scientific Inc, USA) following the manufacturer’s protocol. Lipofectamine 2000 was used in this study, and larvae and nymphs were tested. P0 Cy3-labeled dsRNA was soaked in Lipofectamine 2000 (1 μg/μl) for 24 h. Then, the ticks were washed three times in 1 × PBS in a dark room and observed through an inverted fluorescence microscope. P0 Cy3-labeled dsRNA in RNase free water (1 μg/μl) was used as the control.

### 2.7 Effect of soaking on different time periods

Lipofectamine 2000 was used with larval, nymphal and adult ticks. P0 dsRNA was soaked in Lipofectamine 2000 (1 μg/μl) for 3, 6, 12 and 24 h. Then, ticks were washed and rested for 5 d before RNA was extracted for real-time PCR analysis. Unsoaked ticks were used as the control. Each test was repeated three times.

### 2.8 Effects of different dsRNA concentrations

Lipofectamine 2000 was used with larval, nymphal and adult ticks. Different concentrations of P0 dsRNA (0.5, 1 and 2 μg/μl) were soaked in Lipofectamine 2000 for 24 h. Then, ticks were washed and rested for 5 d before RNA was extracted for real-time PCR analysis. The ticks treated with Lipofectamine 2000 without P0 dsRNA were used as the control. Each test was repeated three times.

### 2.9 Tick bioassays

Nymph and adult ticks were tested in this experiment. dsRNAs of tick P0 and luciferase genes (control) were separately soaked in Lipofectamine 2000 (1 μg/μl) for 12 h. Then, ticks were stored at 25 °C for 1 d in a moist chamber. The numbers of dead ticks were recorded, and the live ticks were allowed to feed on rabbits. Each group of ticks (P0 and control) was allowed to feed on three rabbits (50 nymphs on the right ear; 30 adult females with 20 adult males on the left ear). A successful tick feeding was determined by measuring the attachment rate after 24 h, the engorgement rate, and the molting rate of nymphs and oviposition rate of adult females.

The attachment rate was calculated as the percentage of attached ticks (nymphs or adult females) against all nymphs or adult females applied to the ear bag, while the engorgement rate was considered as the percent of engorged (nymphs or adult female) ticks compared with all ticks (nymph or adult females) applied to the ear bag. On the other hand, molting rate for nymphs was calculated by comparing the number of newly molted adult ticks against the number of all engorged nymphs. Lastly, the rate of oviposition was defined as the number of egg laying engorged females compared with the number of all engorged female ticks.

### 2.10 Statistical analyses

SPSS software was used for the statistical analyses. All data represent the mean ± SEM of each experiment. Statistically significant differences were examined between test groups and the control. Significant difference (P < .05) as calculated by Student’s *t*-test.

## 3 Results

### 3.1 Cloning and sequence analysis of the *R. haemaphysaloides* P0 gene

**Fig. 1:**
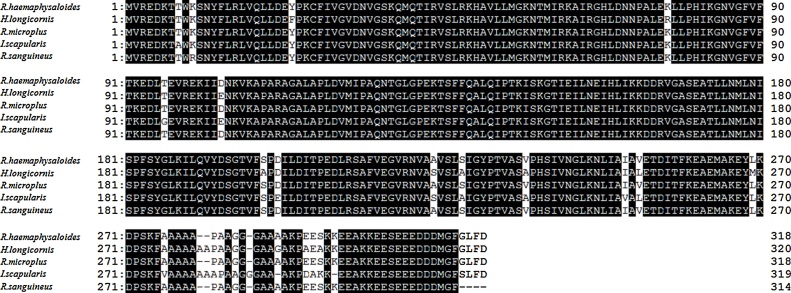
Alignment of the deduced protein sequences of P0 from *R. haemaphysaloides* and other tick species. Identical residues are shaded black. The protein sequence accession numbers for *H. longicornis*, *R. microplus*, *I. scapularis*, *R. sanguineus* and *R. haemaphysaloides* are ABW16870.1, AGQ49465.1, AAY66850.1, AJQ18906.1 and ALJ02546.1, respectively.

The full-length cDNA of P0 gene from *R. haemaphysaloides* obtained by RACE is 1138 bp long [GenBank: KR697563]. The ORF of the cDNA sequence encodes a putative protein with 318 amino acids, with a predicted molecular mass of 35 kDa. A database search using the BLAST program revealed a high sequence similarity between the *R. haemaphysaloides*’ P0 protein and P0 proteins from various tick species. The alignment of the deduced P0 protein sequence (ALJ02546.1) with those from *Haemaphysalis longicornis* (ABW16870.1), *Rhipicephalus microplus* (AGQ49465.1), *Ixodes scapularis* (AAY66850.1), and *Rhipicephalus sanguineus* (AJQ18906.1) yielded high amino acid identities of 95%–99% (Fig. 1). This result indicated that the P0 gene is highly conserved in different ticks.

### 3.2 dsRNA synthesis

Primers were designed and synthesized to contain the T7 promoter sequence. PCR products (600 bp and 736 bp) were gel-purified and used to synthesize luciferase and P0 dsRNA, respectively. Integrity of the dsRNA was checked on 1% agarose gels. The dsRNA was quantified at 260 nm using a spectrophotometer. About 5–8 μg/μl dsRNA in 50 μl RNase free water was used in a reaction.

### 3.3 Gene silencing efficiency

**Fig. 2:**
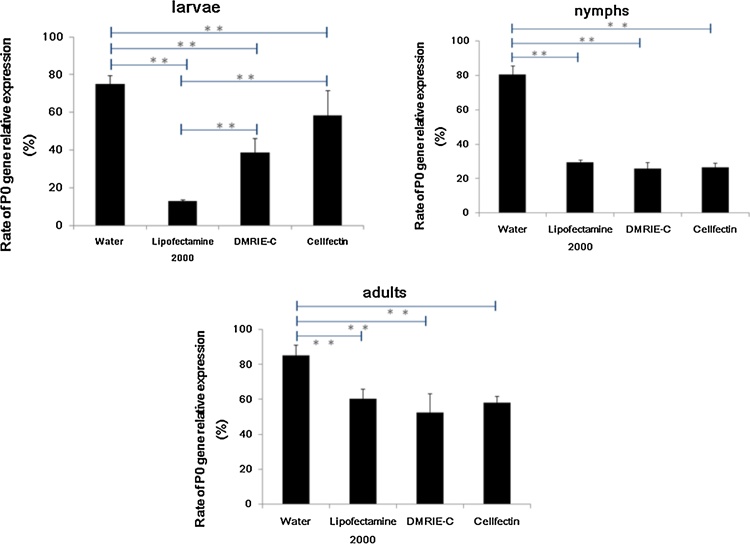
P0 gene silencing *via* dsRNA by soaking with different liposomes and water. *R. haemaphysaloides* ticks were soaked for 24 h by setting a tube on a vertical rotating device at room temperature. After soaking, ticks were rinsed in distilled water and dried on paper towels. They were then allowed to rest for 5 d before RNA was extracted for real-time PCR analysis. Each test was repeated three times. Relative expression of P0 against the tick elongation factor-1α was calculated using the ticks soaked in water without P0 dsRNA as the control. Significant differences between different groups are marked with asterisks (p < .01).

Three kinds of lipid agents, Lipofectamine 2000, DMRIE-C and Cellfectin, were used to mediate P0 dsRNA delivery and to determine silencing efficiency using quantitative PCR. A water control without a liposome agent was included under the same conditions. The results are shown in Fig. 2. All liposome agents helped mediate the P0 dsRNA delivery, and the target gene silencing increased significantly compared with the water control. There were no significant differences among the three liposomes in nymphs and adult ticks; however, Lipofectamine 2000 significantly increased gene silence compared with the other two liposomes in larvae. This result showed that liposomes could mediate the delivery of dsRNA into ticks and silence the target gene *via* body surface absorption.

### 3.4 Observation of Cy3-labeled dsRNA in *R. haemaphysaloides* after soaking

**Fig. 3:**
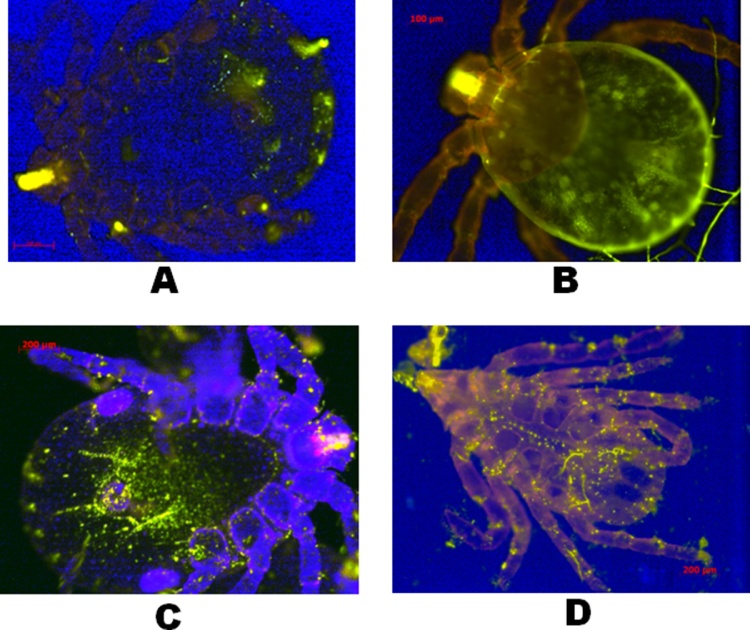
Observation of Cy3-labeled dsRNA in *R. haemaphysaloides* after soaking in Lipofectamine 2000 and water. After soaking, the *R. haemaphysaloides* ticks were washed three times in PBS in dark and observed through an inverted fluorescence microscope. The red fluorescence was transformed into green for a clearer image. Cy3-labeled P0 dsRNA in RNase free water was used as the control. A, larvae soaked in labeled dsRNA in water. B, larvae soaked in labeled dsRNA in Lipofectamine 2000. C, nymphs soaked in labeled dsRNA in water. D, nymphs soaked in labeled dsRNA in Lipofectamine 2000. (For interpretation of the references to color in this figure legend, the reader is referred to the web version of this article.)

P0 dsRNA delivery was observed using fluorescence-labeling. After optimal soaking conditions, ticks were washed in 1 × PBS and observed through an inverted fluorescence microscope. Labeled dsRNA presented as punctuate distributed red fluorescence *in vivo* in the experimental group, the red fluorescence was transformed into green to obtain clearer images (Fig. 3B). These results showed the delivery of dsRNA into the tick *via* body surface absorption. Moreover, dsRNA molecules also entered the tick body through the mouth, pore canals and canaliculi that open externally to the outer epicuticle (Fig. 3A). More effective delivery through the liposome was confirmed compared to that of water (B, D > A, C).

### 3.5 Effect of soaking time on P0 gene silencing efficiency

**Fig. 4:**
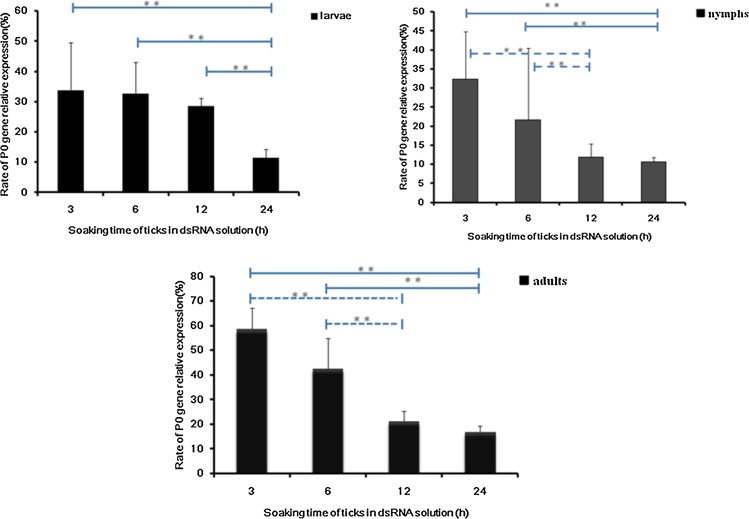
Effects of soaking time on gene silencing. The *R. haemaphysaloides* larvae, nymph and adult ticks were soaked in Lipofectamine 2000 with P0 dsRNA for 3, 6, 12 and 24 h. Relative expression of the P0 gene against the tick elongation factor-1α gene was calculated using the 2^−△△Ct^ method using the unsoaked ticks as controls. Significant statistical differences between different soaking times are marked with two asterisks (p < .01).

To determine the effect of soaking time on gene silencing, P0 dsRNA was soaked in Lipofectamine 2000 (1 μg/μl) for 3, 6, 12 and 24 h. RNAi effects were tested using real-time PCR, and the results are shown in Fig. 4. The target gene expression level decreased as the soaking time increased. In larvae, there was a significant decrease after 24 h of soaking compared with 12, 6 and 3 h. However, in nymphs and adults, there was a significant decrease in expression after 12 h of soaking.

### 3.6 Effect of dsRNA concentrations on P0 gene silencing efficiency

**Fig. 5:**
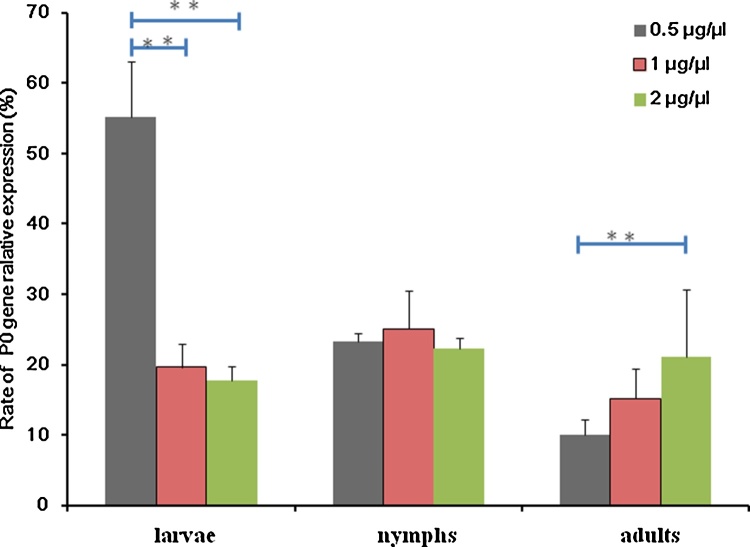
Effects of dsRNA concentrations on gene silencing. *R. haemaphysaloides* ticks were soaked in different concentrations of P0 dsRNA (0.5, 1 and 2 μg/μl) in Lipofectamine 2000 for 24 h. Then, ticks were washed and rested for 5 d before extracting RNA for real-time PCR analysis. The relative expression of P0 gene was calculated using the tick elongation factor-1α as the reference gene and the ticks soaked in Lipofectamine 2000 solution without P0 dsRNA as the control. Significant differences between the different groups are marked with two asterisks (p < .01).

To determine the effect of dsRNA concentrations on gene silencing, P0 dsRNA (0.5, 1 and 2 μg/μl) was soaked in Lipofectamine 2000 for 24 h. RNAi effects were tested using quantitative PCR, and the results are shown in Fig. 5. In larvae, there was a significant difference between 0.5 μg/μl group and 1 or 2 μg/μl group; for nymphs, there was no significant difference among the three different concentrations; and for adults, the lower concentration was better in silencing efficiency. Overall, the target gene expression was not dose-dependent.

### 3.7 Tick bioassays to determine the feeding, molting and reproductive rates after soaking

Nymphs and adults died during the course of this treatment. In nymphs, compared with the luciferase dsRNA control group, there was a significant decrease in the molting rate in the P0 dsRNA-treated group (Table 1). In adult ticks, compared with the luciferase dsRNA control group, there were significant decreases (P < .01) in the engorgement rate and oviposition rate in the P0 dsRNA treatment group (Table 2).

**Table 1:** Effects of dsRNA treatment on *R. haemaphysaloides* nymphal tick blood feeding.^a^

Groups	Death rate after soaking (%)	Attachment rate at 24 h (%)	Engorgement rate (%)	Molting rate (%)
P0	2.9 ± 0.7	89.5 ± 2.3	86.6 ± 5.3	12.3 ± 0.6^b^
Control (luciferase)	2.7 ± 0.5	85.9 ± 2.9	89.9 ± 1.9	89.6 ± 4.2

a Values are expressed as averages ± standard error.

b Significant difference (P < 0.05) as calculated by Student’s *t*-test.

**Table 2:** Effects of dsRNA treatment on *R. haemaphysaloides* adult female tick blood feeding.^a^

Groups	Death rate after soaking (%)	Attachment rate at 24 h (%)	Engorgement rate (%)	Oviposition rate (%)
P0	4.3 ± 0.6	91.3 ± 1.7	68.3 ± 3.7^b^	6.7 ± 0.6^b^
Control (luciferase)	4.6 ± 0.3	88.9 ± 5.1	87.8 ± 5.1	91.0 ± 6.3

a Values were expressed as averages ± standard error.

b Significant difference (P < 0.05) as calculated by Student’s *t*-test.

## 4 Discussion

A group of acidic proteins in the Eukaryotic ribosomes, which form a stalk-like structure in the largest ribosome subunit are named P proteins (P0–P2) due to their ability to be phosphorylated. They play a fundamental role in regulating translational activity of ribosomes. P0 binds directly to P1, P2, 28S rRNA and the factor eEF2, and its absence leads to loss of protein synthesis, and cell death (Wojda et al., 2002). A small body of work targeting ribosomal transcripts indicates that they may be attractive targets for RNAi-mediated knockdown because of the importance of the ribosome in most cellular processes.

In *Drosophila melanogaster*, mutated ribosomal proteins inhibited oviposition in adults or induced mortality in larvae among other abnormalities (Kay and Jacobs-Lorena, 1987; Cramton and Laski, 1994), while in *R. microplus* knocking down ribosomal transcripts led to a reduction in oviposition (Kurscheid et al., 2009). Micro-injection of P0 specific dsRNA in *H. longicornis* ticks showed a mortality rate of 96%, suggesting that P0 ribosomal protein is necessary for blood feeding and viability in ticks (Gong et al., 2008). In this study, the cDNA of P0 gene from the tick, *R. haemaphysaloides*, was cloned and analyzed revealing highly conserved regions when compared to other tick species. The highly conserved nature of the P0 gene suggests its potential as an anti-tick control agent against multiple tick species.

The soaking technique for RNAi was first introduced in nematodes, and then it was applied in insect studies. Despite the presence of the exoskeleton, uptake of the dsRNA through the whole body of an insect was found possible. Direct spray of dsRNA on newly hatched *Ostrinia furnacalis* larvae resulted in considerable mortality ranging from 40% to 100% after treatment as verified by qPCR (Wang et al., 2011). It has also been found that immersion of whole tick in dsRNA dissolved in water can induce gene silencing (Galay et al., 2016). To promote the use of dsRNA as an anti-tick control agent, a method that specifically modifies/coats the dsRNA to enhance its uptake by the tick body and enhances efficiency of gene silencing needs to be developed. A good example is to coat with liposomes to deliver siRNA to mammalian cells, specific tissues and some insects (Kurreck, 2009; Whyard et al., 2009). Liposomes containing dsRNA delivery systems have been explored in insects, to prevent RNAi trigger degradation when used in field applications (Whyard et al., 2015; Van Ekert et al., 2014). Spraying dsRNA would be possible if they were properly protected by a coating.

In this study, to confirm that P0 dsRNA was successfully absorbed through the body surface by soaking in liposomes containing the target dsRNA, P0 dsRNA was labeled with Cy3 dye. After soaking; ticks were examined and the results showed that the dsRNA was successfully taken up through the body surface of *R. haemaphysaloides*. Punctuate distribution of dsRNA in the tick body was a previously described feature (Karim et al., 2010) using electroporation technique. We found that the liposome mediated dsRNA delivery for P0 showed better uptake efficiency than that of naked dsRNA. The exact entry site of dsRNA into the tick’s body is still unknown. However, we speculate that like acaricides, dsRNA molecules may penetrate the cuticle through the pore canals and canaliculi that open externally to the outer epicuticle (Sonenshine, 1991).

Compared with other blood sucking arthropods, the feeding time of hard ticks is particularly long and usually takes 4–14 days depending on the species and developmental stage (Kaufman, 2007). Tick’s long feeding duration on mammalian hosts is an important factor in determining the safety of an anti-tick agent control. As a potential bio-insecticide, dsRNA application needs to address issues like environmental pollution. However, in mammalian cells, it has been observed that dsRNAs longer than ∼30 nt elicit an antiviral associated interferon response, which ultimately results in non-specific suppression of gene expression through activation of RNase L and general degradation of RNA molecules (Stark et al., 1998). Therefore, long dsRNAs is safe to tick mammalian hosts, suggesting that long dsRNAs can be a potential strategy for tick control in the field.

In this study, the P0 dsRNAs from *R. haemaphysaloides* was used to evaluate its potential as an anti-tick control agent and its role on feeding, molting or reproduction. In these preliminary assays, soaking time was found to be the most important factor in dsRNA delivery and gene silencing compared with liposome types and dsRNA concentration. The optimum immersion conditions for all developmental stages of ticks should be determined in further studies. Although there are some limitations on its cost and environment risks, strategies for insecticide delivery could be adapted to function as RNAi trigger delivery systems and thereby expedite transformation of RNAi technology from the laboratory to the field. We conclude that anti-tick agents based on dsRNAs might have potential to control tick populations.
